# 1-Benzyl-6-chloro­indoline-2,3-dione

**DOI:** 10.1107/S1600536811048665

**Published:** 2011-11-25

**Authors:** Hua-quan Liu, Dong-mei Fan, De-cai Wang, Ping-Kai Ou-yang

**Affiliations:** a Sate Key Laboratory of Materials-Oriented Chemcial Engineering, College of Life Science and Pharmaceutical Engineering, Nanjing University of Technology, Xinmofan Road No. 5 Nanjing, Nanjing 210009, People’s Republic of China

## Abstract

In the title compound, C_15_H_10_ClNO_2_,the dihedral angle between the mean planes of the benzene and 6-chloro­indoline-2,3-dione ring systems, linked through a methyl­ene group, is 81.68 (10)°. In the crystal, mol­ecules are connected by C—H⋯O hydrogen bonds, generating *C*(6) chains propagating in [010].

## Related literature

For general background to isatin derivatives, see: Vine *et al.* (2007 ▶); Matesic *et al.* (2008 ▶). For further synthetic details, see: Bouhfid *et al.* (2005 ▶).
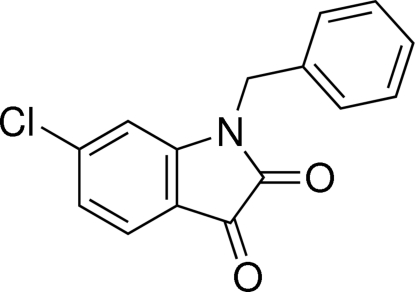


## Experimental

### 

#### Crystal data

C_15_H_10_ClNO_2_
*M* *_r_* = 271.69
Triclinic, 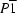

*a* = 7.1870 (14) Å
*b* = 7.5800 (15) Å
*c* = 12.012 (2) Å
α = 80.24 (3)°
β = 84.90 (3)°
γ = 79.74 (3)°
*V* = 633.4 (2) Å^3^
*Z* = 2
Mo *K*α radiation
μ = 0.30 mm^−1^
*T* = 293 K
0.30 × 0.20 × 0.10 mm

#### Data collection

Enraf–Nonius CAD-4 diffractometer
Absorption correction: ψ scan (North *et al.*, 1968 ▶) *T*_min_ = 0.916, *T*_max_ = 0.971
2520 measured reflections
2322 independent reflections
1837 reflections with *I* > 2σ(*I*)
*R*_int_ = 0.015
3 standard reflections every 200 reflections  intensity decay: 1%

#### Refinement

*R*[*F* ^2^ > 2σ(*F* ^2^)] = 0.045
*wR*(*F* ^2^) = 0.155
*S* = 1.00
2322 reflections
172 parameters
1 restraint
H-atom parameters constrained
Δρ_max_ = 0.19 e Å^−3^
Δρ_min_ = −0.25 e Å^−3^

### 

Data collection: *CAD-4 EXPRESS* (Enraf–Nonius, 1994 ▶); cell refinement: *CAD-4 EXPRESS*; data reduction: *XCAD4* (Harms & Wocadlo, 1995 ▶); program(s) used to solve structure: *SHELXS97* (Sheldrick, 2008 ▶); program(s) used to refine structure: *SHELXL97* (Sheldrick, 2008 ▶); molecular graphics: *SHELXTL* (Sheldrick, 2008 ▶); software used to prepare material for publication: *PLATON* (Spek, 2009 ▶).

## Supplementary Material

Crystal structure: contains datablock(s) global, I. DOI: 10.1107/S1600536811048665/hb6512sup1.cif

Structure factors: contains datablock(s) I. DOI: 10.1107/S1600536811048665/hb6512Isup2.hkl

Supplementary material file. DOI: 10.1107/S1600536811048665/hb6512Isup3.cml

Additional supplementary materials:  crystallographic information; 3D view; checkCIF report

## Figures and Tables

**Table 1:** Hydrogen-bond geometry (Å, °)

*D*—H⋯*A*	*D*—H	H⋯*A*	*D*⋯*A*	*D*—H⋯*A*
C9—H9*A*⋯O1^i^	0.93	2.58	3.431 (3)	152

Symmetry code: (i) 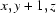.

**Fig. 1.**
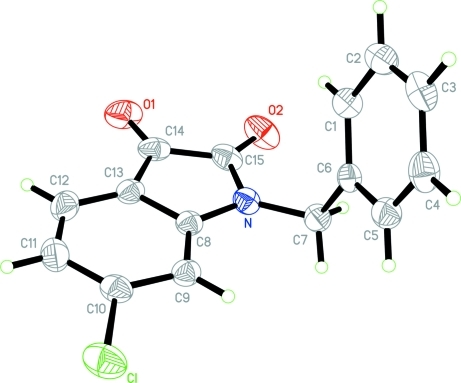
The molecular structure of (I), showing displacement ellipsoids at the 30% probability level.

**Fig. 2.**
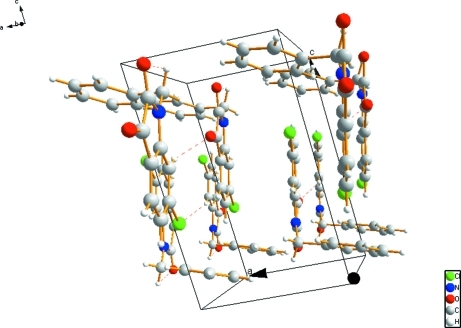
A packing diagram of (I). The intermolecular hydrogen bonds are shown as dashed lines.

## supplementary crystallographic information

### Comment

Halogenated derivatives of isatin have been found to exhibit cytotoxic and antineoplastic activity(Vine *et al.*, 2007; Matesic *et al.*, 2008). As a part of our studies into the synthesis of isatin derivatives, the title compound (I) 1-benzyl-6-chloroindoline-2,3-dione was synthesized (Bouhfid *et al.* (2005)). We report herein its crystal structure.

In the title compound, C_15_H_10_ClNO_2_, the indoline and benzene moieties are linked by a methylene group with a C6—C7(methylene)-N angle of 112.49 (2)° (Fig. 1). The dihedral angle between the mean planes of the benzene and 6-chloroindoline-2,3-dione is 81.68 (10) °. In the crystal structure, C—H···O hydrogen bonds link the molecules (Fig. 2 and Table 1).

### Experimental

Isatin(1.47 g, 0.01 mol) was reacted with benzyl bromide (0.02 mol) in the presence of K_2_CO_3_ (2.76 g, 0.02 mol) and tetrabutylammonium bromide (0.32 g, 0.001 mol) in DMF (60 ml). After 12 h stirring at rt, the precipitate was removed by filtration and purified by recrystallization from ethanol(m.p. 175.2–176.1 °C; yield 70%). The yellow blocks of the title compound were obtained by slow evaporation from ethanol at room temperature.

### Refinement

All H atoms were placed geometrically (C—H = 0.93–0.96 Å) and refined as riding with *U*_iso_(H) = 1.2*U*_eq_(carrier) or 1.5*U*_eq_(methyl carrier).

### Crystal data

**Table:**

C_15_H_10_ClNO_2_	*Z* = 2
*M**_r_* = 271.69	*F*(000) = 280
Triclinic, *P*1	*D*_x_ = 1.424 Mg m^−^^3^
Hall symbol: -P 1	Mo *K*α radiation, λ = 0.71073 Å
*a* = 7.1870 (14) Å	Cell parameters from 25 reflections
*b* = 7.5800 (15) Å	θ = 10–13°
*c* = 12.012 (2) Å	µ = 0.30 mm^−^^1^
α = 80.24 (3)°	*T* = 293 K
β = 84.90 (3)°	Block, yellow
γ = 79.74 (3)°	0.30 × 0.20 × 0.10 mm
*V* = 633.4 (2) Å^3^	

### Data collection

**Table:**

Enraf–Nonius CAD-4 diffractometer	1837 reflections with *I* > 2σ(*I*)
Radiation source: fine-focus sealed tube	*R*_int_ = 0.015
graphite	θ_max_ = 25.4°, θ_min_ = 1.7°
ω/2θ scans	*h* = 0→8
Absorption correction: ψ scan (North *et al.*, 1968)	*k* = −8→9
*T*_min_ = 0.916, *T*_max_ = 0.971	*l* = −14→14
2520 measured reflections	3 standard reflections every 200 reflections
2322 independent reflections	intensity decay: 1%

### Refinement

**Table:**

Refinement on *F*^2^	Primary atom site location: structure-invariant direct methods
Least-squares matrix: full	Secondary atom site location: difference Fourier map
*R*[*F*^2^ > 2σ(*F*^2^)] = 0.045	Hydrogen site location: inferred from neighbouring sites
*wR*(*F*^2^) = 0.155	H-atom parameters constrained
*S* = 1.00	*w* = 1/[σ^2^(*F*_o_^2^) + (0.1*P*)^2^ + 0.160*P*] where *P* = (*F*_o_^2^ + 2*F*_c_^2^)/3
2322 reflections	(Δ/σ)_max_ < 0.001
172 parameters	Δρ_max_ = 0.19 e Å^−^^3^
1 restraint	Δρ_min_ = −0.25 e Å^−^^3^

### Special details

**Table:**

Geometry. All e.s.d.'s (except the e.s.d. in the dihedral angle between two l.s. planes) are estimated using the full covariance matrix. The cell e.s.d.'s are taken into account individually in the estimation of e.s.d.'s in distances, angles and torsion angles; correlations between e.s.d.'s in cell parameters are only used when they are defined by crystal symmetry. An approximate (isotropic) treatment of cell e.s.d.'s is used for estimating e.s.d.'s involving l.s. planes.
Refinement. Refinement of *F*^2^ against ALL reflections. The weighted *R*-factor *wR* and goodness of fit *S* are based on *F*^2^, conventional *R*-factors *R* are based on *F*, with *F* set to zero for negative *F*^2^. The threshold expression of *F*^2^ > σ(*F*^2^) is used only for calculating *R*-factors(gt) *etc*. and is not relevant to the choice of reflections for refinement. *R*-factors based on *F*^2^ are statistically about twice as large as those based on *F*, and *R*- factors based on ALL data will be even larger.

### Fractional atomic coordinates and isotropic or equivalent isotropic displacement parameters (Å^2^)

**Table:**

	*x*	*y*	*z*	*U*_iso_*/*U*_eq_	
Cl	0.15893 (11)	0.74198 (12)	0.63411 (6)	0.0780 (3)	
N	0.3532 (3)	0.5031 (3)	0.25370 (16)	0.0484 (5)	
O1	0.3966 (3)	0.0350 (3)	0.3312 (2)	0.0835 (7)	
C1	0.0178 (3)	0.6495 (3)	0.1103 (2)	0.0534 (6)	
H1A	0.0667	0.5310	0.0999	0.064*	
O2	0.4404 (3)	0.3028 (3)	0.12621 (18)	0.0805 (7)	
C2	−0.1670 (4)	0.7221 (4)	0.0844 (2)	0.0599 (7)	
H2A	−0.2413	0.6526	0.0567	0.072*	
C3	−0.2404 (4)	0.8970 (4)	0.0998 (2)	0.0608 (7)	
H3A	−0.3644	0.9461	0.0823	0.073*	
C4	−0.1308 (4)	0.9994 (4)	0.1409 (2)	0.0640 (7)	
H4A	−0.1809	1.1177	0.1515	0.077*	
C5	0.0548 (4)	0.9268 (3)	0.1666 (2)	0.0562 (6)	
H5A	0.1283	0.9973	0.1942	0.067*	
C6	0.1314 (3)	0.7513 (3)	0.15178 (18)	0.0446 (5)	
C7	0.3348 (3)	0.6736 (3)	0.1755 (2)	0.0514 (6)	
H7A	0.4057	0.6538	0.1049	0.062*	
H7B	0.3896	0.7609	0.2071	0.062*	
C8	0.3061 (3)	0.4880 (3)	0.37094 (19)	0.0432 (5)	
C9	0.2568 (3)	0.6255 (3)	0.4348 (2)	0.0477 (6)	
H9A	0.2489	0.7470	0.4027	0.057*	
C10	0.2197 (3)	0.5730 (4)	0.5496 (2)	0.0528 (6)	
C11	0.2296 (4)	0.3939 (4)	0.6003 (2)	0.0634 (7)	
H11A	0.2030	0.3650	0.6778	0.076*	
C12	0.2797 (4)	0.2591 (4)	0.5343 (3)	0.0622 (7)	
H12A	0.2875	0.1378	0.5668	0.075*	
C13	0.3180 (3)	0.3053 (3)	0.4202 (2)	0.0502 (6)	
C14	0.3748 (3)	0.1993 (3)	0.3277 (2)	0.0582 (7)	
C15	0.3958 (3)	0.3341 (3)	0.2226 (2)	0.0569 (7)	

### Atomic displacement parameters (Å^2^)

**Table:**

	*U*^11^	*U*^22^	*U*^33^	*U*^12^	*U*^13^	*U*^23^
Cl	0.0726 (5)	0.1012 (7)	0.0686 (5)	−0.0153 (4)	0.0032 (4)	−0.0405 (4)
N	0.0482 (11)	0.0473 (11)	0.0489 (11)	0.0000 (8)	−0.0083 (9)	−0.0108 (8)
O1	0.0867 (15)	0.0451 (11)	0.1232 (19)	0.0015 (10)	−0.0367 (13)	−0.0231 (11)
C1	0.0524 (14)	0.0538 (14)	0.0537 (14)	−0.0048 (11)	−0.0034 (11)	−0.0114 (11)
O2	0.0847 (14)	0.0836 (15)	0.0721 (14)	0.0207 (11)	−0.0193 (11)	−0.0381 (11)
C2	0.0532 (15)	0.0760 (18)	0.0523 (15)	−0.0125 (13)	−0.0058 (11)	−0.0116 (12)
C3	0.0512 (14)	0.0773 (18)	0.0454 (13)	0.0050 (13)	−0.0029 (11)	−0.0023 (12)
C4	0.0730 (18)	0.0544 (15)	0.0553 (15)	0.0097 (13)	−0.0018 (13)	−0.0051 (12)
C5	0.0682 (16)	0.0525 (14)	0.0487 (14)	−0.0091 (12)	−0.0068 (12)	−0.0094 (11)
C6	0.0498 (13)	0.0476 (13)	0.0342 (11)	−0.0078 (10)	−0.0002 (9)	−0.0016 (9)
C7	0.0474 (13)	0.0574 (14)	0.0489 (13)	−0.0112 (11)	−0.0016 (10)	−0.0055 (11)
C8	0.0347 (11)	0.0441 (12)	0.0510 (13)	−0.0048 (9)	−0.0096 (9)	−0.0066 (9)
C9	0.0441 (12)	0.0449 (12)	0.0550 (14)	−0.0073 (10)	−0.0075 (10)	−0.0077 (10)
C10	0.0415 (12)	0.0674 (16)	0.0533 (14)	−0.0119 (11)	−0.0056 (10)	−0.0156 (12)
C11	0.0532 (15)	0.083 (2)	0.0517 (15)	−0.0192 (13)	−0.0068 (12)	0.0057 (13)
C12	0.0577 (15)	0.0552 (15)	0.0711 (18)	−0.0142 (12)	−0.0175 (13)	0.0090 (13)
C13	0.0419 (12)	0.0435 (13)	0.0656 (16)	−0.0075 (9)	−0.0162 (11)	−0.0027 (11)
C14	0.0484 (13)	0.0441 (13)	0.0858 (19)	0.0003 (10)	−0.0270 (13)	−0.0175 (12)
C15	0.0471 (13)	0.0589 (15)	0.0652 (16)	0.0085 (11)	−0.0178 (11)	−0.0216 (12)

### Geometric parameters (Å, °)

**Table:**

Cl—C10	1.739 (3)	C5—H5A	0.9300
N—C15	1.370 (3)	C6—C7	1.509 (3)
N—C8	1.409 (3)	C7—H7A	0.9700
N—C7	1.456 (3)	C7—H7B	0.9700
O1—C14	1.221 (3)	C8—C9	1.375 (3)
C1—C2	1.385 (4)	C8—C13	1.402 (3)
C1—C6	1.391 (3)	C9—C10	1.385 (4)
C1—H1A	0.9300	C9—H9A	0.9300
O2—C15	1.224 (3)	C10—C11	1.383 (4)
C2—C3	1.374 (4)	C11—C12	1.377 (4)
C2—H2A	0.9300	C11—H11A	0.9300
C3—C4	1.372 (4)	C12—C13	1.372 (4)
C3—H3A	0.9300	C12—H12A	0.9300
C4—C5	1.390 (4)	C13—C14	1.467 (4)
C4—H4A	0.9300	C14—C15	1.498 (3)
C5—C6	1.381 (3)		
			
C15—N—C8	110.1 (2)	H7A—C7—H7B	107.8
C15—N—C7	125.0 (2)	C9—C8—C13	121.4 (2)
C8—N—C7	124.52 (19)	C9—C8—N	128.0 (2)
C2—C1—C6	121.0 (2)	C13—C8—N	110.6 (2)
C2—C1—H1A	119.5	C8—C9—C10	116.3 (2)
C6—C1—H1A	119.5	C8—C9—H9A	121.9
C3—C2—C1	119.8 (3)	C10—C9—H9A	121.9
C3—C2—H2A	120.1	C11—C10—C9	123.5 (2)
C1—C2—H2A	120.1	C11—C10—Cl	118.5 (2)
C4—C3—C2	120.0 (2)	C9—C10—Cl	118.0 (2)
C4—C3—H3A	120.0	C12—C11—C10	118.9 (3)
C2—C3—H3A	120.0	C12—C11—H11A	120.5
C3—C4—C5	120.1 (3)	C10—C11—H11A	120.5
C3—C4—H4A	119.9	C13—C12—C11	119.4 (2)
C5—C4—H4A	119.9	C13—C12—H12A	120.3
C6—C5—C4	120.7 (3)	C11—C12—H12A	120.3
C6—C5—H5A	119.6	C12—C13—C8	120.4 (2)
C4—C5—H5A	119.6	C12—C13—C14	133.5 (2)
C5—C6—C1	118.2 (2)	C8—C13—C14	106.1 (2)
C5—C6—C7	121.1 (2)	O1—C14—C13	128.7 (3)
C1—C6—C7	120.6 (2)	O1—C14—C15	125.0 (3)
N—C7—C6	112.49 (19)	C13—C14—C15	106.3 (2)
N—C7—H7A	109.1	O2—C15—N	125.5 (2)
C6—C7—H7A	109.1	O2—C15—C14	127.6 (2)
N—C7—H7B	109.1	N—C15—C14	107.0 (2)
C6—C7—H7B	109.1		
			
C6—C1—C2—C3	−0.1 (4)	Cl—C10—C11—C12	−179.28 (19)
C1—C2—C3—C4	−0.1 (4)	C10—C11—C12—C13	−0.1 (4)
C2—C3—C4—C5	0.3 (4)	C11—C12—C13—C8	−0.1 (4)
C3—C4—C5—C6	−0.2 (4)	C11—C12—C13—C14	179.6 (2)
C4—C5—C6—C1	0.1 (4)	C9—C8—C13—C12	0.2 (3)
C4—C5—C6—C7	177.8 (2)	N—C8—C13—C12	179.4 (2)
C2—C1—C6—C5	0.1 (4)	C9—C8—C13—C14	−179.6 (2)
C2—C1—C6—C7	−177.6 (2)	N—C8—C13—C14	−0.4 (2)
C15—N—C7—C6	97.7 (3)	C12—C13—C14—O1	2.7 (5)
C8—N—C7—C6	−73.8 (3)	C8—C13—C14—O1	−177.6 (2)
C5—C6—C7—N	126.2 (2)	C12—C13—C14—C15	−179.3 (3)
C1—C6—C7—N	−56.1 (3)	C8—C13—C14—C15	0.4 (2)
C15—N—C8—C9	179.4 (2)	C8—N—C15—O2	179.3 (2)
C7—N—C8—C9	−8.0 (3)	C7—N—C15—O2	6.8 (4)
C15—N—C8—C13	0.3 (3)	C8—N—C15—C14	0.0 (3)
C7—N—C8—C13	172.9 (2)	C7—N—C15—C14	−172.6 (2)
C13—C8—C9—C10	−0.1 (3)	O1—C14—C15—O2	−1.5 (4)
N—C8—C9—C10	−179.1 (2)	C13—C14—C15—O2	−179.5 (3)
C8—C9—C10—C11	−0.1 (4)	O1—C14—C15—N	177.8 (2)
C8—C9—C10—Cl	179.39 (16)	C13—C14—C15—N	−0.2 (3)
C9—C10—C11—C12	0.3 (4)		

### Hydrogen-bond geometry (Å, °)

**Table:**

*D*—H···*A*	*D*—H	H···*A*	*D*···*A*	*D*—H···*A*
C9—H9A···O1^i^	0.93	2.58	3.431 (3)	152

Symmetry codes: (i) *x*, *y*+1, *z*.
